# A newly discovered neural stem cell population is generated by the optic lobe neuroepithelium during embryogenesis in *Drosophila melanogaster*

**DOI:** 10.1242/dev.166207

**Published:** 2018-09-25

**Authors:** Anna E. Hakes, Leo Otsuki, Andrea H. Brand

**Affiliations:** The Gurdon Institute and Department of Physiology, Development and Neuroscience, University of Cambridge, Tennis Court Road, Cambridge CB2 1QN, UK

**Keywords:** Neural stem cell, Neuroepithelium, Neuroblast, Stem cell divisions, Symmetric/Asymmetric division, Brain

## Abstract

Neural stem cells must balance symmetric and asymmetric cell divisions to generate a functioning brain of the correct size. In both the developing *Drosophila* visual system and mammalian cerebral cortex, symmetrically dividing neuroepithelial cells transform gradually into asymmetrically dividing progenitors that generate neurons and glia. As a result, it has been widely accepted that stem cells in these tissues switch from a symmetric, expansive phase of cell divisions to a later neurogenic phase of cell divisions. In the *Drosophila* optic lobe, this switch is thought to occur during larval development. However, we have found that neuroepithelial cells start to produce neuroblasts during embryonic development, demonstrating a much earlier role for neuroblasts in the developing visual system. These neuroblasts undergo neurogenic divisions, enter quiescence and are retained post-embryonically, together with neuroepithelial cells. Later in development, neuroepithelial cells undergo further cell divisions before transforming into larval neuroblasts. Our results demonstrate that the optic lobe neuroepithelium gives rise to neurons and glia over 60 h earlier than was thought previously.

## Abstract (precis)

**Highlighted Article:** Two types of neural stem cell, with distinct proliferation modes, act side by side to generate the *Drosophila* optic lobe, challenging current dogma that they act sequentially.

## INTRODUCTION

Neural stem cells in the developing brain must regulate their proliferation precisely to generate a functional nervous system. An imbalance between symmetric and asymmetric stem cell divisions can lead to the inadequate production of differentiated progeny or, conversely, to tumour formation. Importantly, work in *Drosophila* has shown that specific brain tumours arise from the mis-regulation of distinct populations of neural stem cells. In *brain tumour* (*brat*) mutants, asymmetrically dividing Type II neuroblasts generate aberrant lineages, whereas symmetrically dividing neuroepithelial cells are the tumour cells of origin in *lethal(3)malignant brain tumour* [*l(3)mbt*] mutants (Bowman et al., 2008; Richter et al., 2011). Thus, identifying different types of neural stem cells and their functions is central to understanding both normal brain development and the diverse causes of tumourigenesis.

The *Drosophila* optic lobe, which forms the visual processing system of the adult brain, is an established system for studying neural stem cells *in vivo* (Egger et al., 2011). The development of the medulla, the largest visual ganglion, shares many parallels with the development of the mammalian cerebral cortex (Brand and Livesey, 2011; Egger et al., 2011). In both tissues, symmetrically dividing neural stem cells (neuroepithelial cells) expand the stem cell pool before transforming into asymmetrically dividing neural stem cells (also called neuroblasts in *Drosophila*) that produce neurons and glia (Fig. S1A) (Egger et al., 2007; Noctor et al., 2004). Previous studies of neuroepithelial cells and neuroblasts in the optic lobe have focussed largely on larval stages (Egger et al., 2011, 2010; Yasugi et al., 2010, 2008). Neuroepithelial cells divide symmetrically in the early larva before a proneural wave sweeps across the neuroepithelium at mid-larval stages, converting neuroepithelial cells into neuroblasts (Yasugi et al., 2008). Here, we demonstrate that this transition begins much earlier, and that neuroepithelial cells and neuroblasts co-exist from embryonic stages.

## RESULTS AND DISCUSSION

### Neuroepithelial cells divide in the embryo

**Fig. 1.**
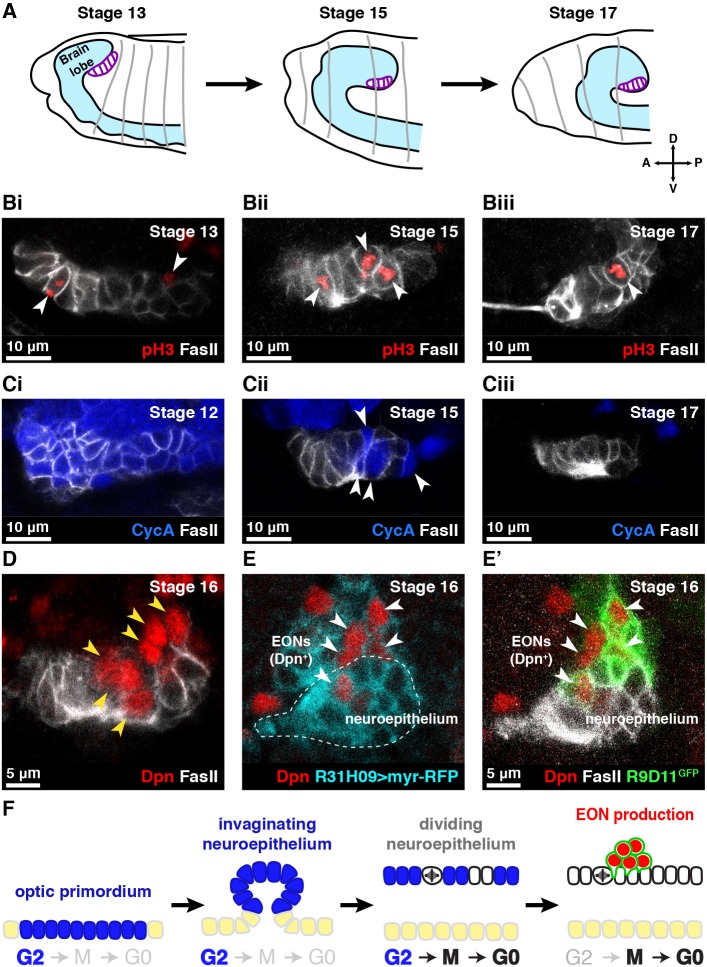
**Embryonic neuroepithelial cells divide and generate EONs.** (A) Schematic depicting the position of the neuroepithelium (purple) as it invaginates from the head ectoderm and attaches to the side of the brain lobe in the embryo. A, anterior; P, posterior; D, dorsal; V, ventral. (Bi-iii) Neuroepithelial cells (FasII^+^, white) co-stained for the mitosis marker pH3 (red) at the indicated embryonic stages. Arrowheads indicate dividing neuroepithelial cells. (Ci-iii) Neuroepithelial cells (white) co-stained for the S/G2 cyclin CycA (blue) at the indicated embryonic stages. Neuroepithelial cells lose CycA expression progressively. Arrowheads in Cii indicate individual neuroepithelial cells that express CycA. (D) Dpn^+^ cells (red, arrowed) appear in close proximity to the neuroepithelium (white) during embryogenesis. (E,E′) RFP expressed using R31H09-GAL4 (cyan in E) labels the embryonic neuroepithelium (white in E′). RFP is inherited by neighbouring Dpn^+^ cells (red, arrowed). These Dpn^+^ cells express R9D11-mCD8-GFP (green). (F) EON production from the embryonic neuroepithelium. The optic primordium invaginates while in G2 (CycA^+^, blue) to give rise to the embryonic neuroepithelium. Neuroepithelial cells undergo mitosis once, losing CycA expression, to produce EONs (green and red). Surface ectoderm cells are indicated in yellow. Brain surface is downwards; interior is upwards. (Bi-E′) Single section confocal images.

The optic lobe primordium is first apparent as a dense patch of cells in the head ectoderm of stage 11 embryos (Hartenstein and Campos-Ortega, 1984; Poulsen, 1950; Turner and Mahowald, 1979). These cells undergo four cell divisions before invaginating from the ectoderm as a neuroepithelial sheet and attaching to the lateral surface of the brain between embryonic stages 12 and 13 (Fig. 1A) (Green et al., 1993).

Neuroepithelial cells can be identified by their expression of Fasciclin II (FasII), the orthologue of neural cell adhesion molecule (NCAM) (Grenningloh et al., 1991; Younossi-Hartenstein et al., 1997). To determine the proliferation pattern of neuroepithelial cells in the embryo, we co-stained for FasII and the cell division marker phospho-histone H3 (pH3). We found pH3^+^ neuroepithelial cells at all developmental stages between optic primordium invagination and the end of embryogenesis (Fig. 1Bi-iii and Fig. S1B). Thus, the neuroepithelium divides throughout embryogenesis, in contrast to a previous suggestion that the optic primordium is dormant in the embryo (Green et al., 1993).

Why was the embryonic neuroepithelium suggested to be dormant? BrdU incorporation assays had shown that neuroepithelial cells do not undergo S phase after invagination (Green et al., 1993). We tested the phase of the cell cycle in which neuroepithelial cells reside as they undergo invagination. We assessed expression of Cyclin A (CycA), a G2-phase cyclin protein, and found that neuroepithelial cells were all CycA^+^ when they invaginated from the ectoderm (Fig. 1Ci). Neuroepithelial cells lost CycA expression over time, concomitant with cell divisions, until they were all CycA^−^ at the end of embryogenesis (Fig. 1Ci-iii). Thus, we found that neuroepithelial cells invaginate in G2 before dividing, explaining both our results and previous observations (Green et al., 1993). As neuroepithelial cells do not undergo S phase in the embryo after invagination (Green et al., 1993), we infer that they divide once each (Fig. 1F).

### The embryonic neuroepithelium generates neuroblasts

We next assessed the role of neuroepithelial cell divisions in the embryo. We found no significant increase in the number of neuroepithelial cells over time (Fig. S1C), indicating that these cell divisions do not serve to increase the size of the neuroepithelium. We therefore tested whether the embryonic neuroepithelium produces neuroblasts, in a similar manner to the late larval neuroepithelium.

We stained for the Hes family transcription factor Deadpan (Dpn), which labels all identified neuroblasts in the *Drosophila* brain (Bier et al., 1992). We found Dpn^+^ cells in close proximity to the neuroepithelium beginning at embryonic stage 12 (Fig. 1D). To test the lineage relationship between neuroepithelial cells and these neuroblasts, we expressed red fluorescent protein (RFP) in the neuroepithelium and assessed whether RFP was inherited by the Dpn^+^ cells. Interestingly, we found that GAL4*^c855a^* and *ogre*-GAL4, two GAL4 drivers that label the larval neuroepithelium (Dillard et al., 2018; Egger et al., 2007), did not express in the embryonic neuroepithelium (data not shown). We therefore identified a GAL4 driver, R31H09-GAL4, that labels the embryonic neuroepithelium (Fig. 1E). When we expressed RFP using R31H09-GAL4, we found that RFP was inherited by the Dpn^+^ cells (Fig. 1E). We conclude that the embryonic neuroepithelium produces neuroblasts, and refer to these neuroblasts as EONs (embryonic optic neuroblasts).

We identified a ∼4 kb fragment of the *earmuff* (*erm*) enhancer (R9D11) that drives expression in EONs consistently, allowing us to track the production of EONs from the embryonic neuroepithelium. (Fig. 1E′). Using R9D11-mCD8-GFP (R9D11 driving expression of membrane-targeted GFP) (Pfeiffer et al., 2008; Zhu et al., 2011), we found that EONs are produced continuously between stage 12 and stage 17 of embryogenesis, with a final number of 8.6±0.7 EONs per brain lobe (Fig. S1D,Ei-iii). EONs were first apparent in the neuroepithelial layer (FasII^+^ Dpn^+^ R9D11^+^) and were extruded medially into the brain, where they downregulated FasII expression (Fig. S1Ei-iii). Importantly, our results demonstrate that neuroepithelial cells produce neuroblasts much earlier (∼60 h earlier) than thought previously (mid-larval stage) (Fig. 1F).

### EONs derive from two spatial domains of the neuroepithelium

We noticed that EONs were generated at specific discontinuous points along the embryonic neuroepithelium. In the larval brain, the neuroepithelium is patterned into spatial domains along the anterior-posterior axis by expression of *Vsx1*, *Optix*, *decapentaplegic* (*dpp*) and *wingless* (*wg*) (Fig. S2A) (Erclik et al., 2008; Gold and Brand, 2014; Kaphingst and Kunes, 1994; reviewed by Bertet, 2017). In addition, the ventral (but not dorsal) half of the neuroepithelium expresses *hedgehog* (*hh*) (Fig. S2A) (Chen et al., 2016; Evans et al., 2009). All spatial domains of the neuroepithelium generate neuroblasts in the larva. As we did not find a continuous band of EONs in the embryo, we reasoned that they might arise from a subset of spatial domains of the neuroepithelium.

**Fig. 2.**
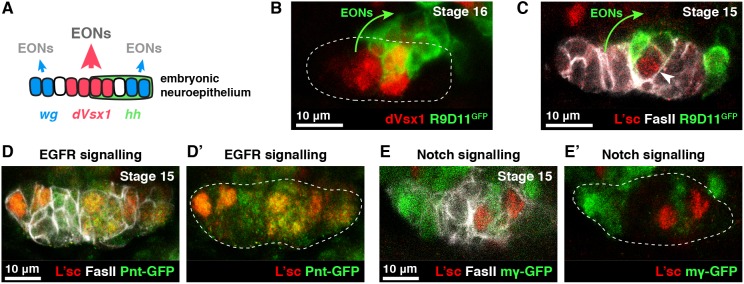
**The embryonic neuroepithelium expresses transition zone markers and produces EONs at specific spatial domains.** (A) Spatial patterning domains in the embryonic neuroepithelium and neuroblast generation (compare with Fig. S2A). The *Vsx1^+^*, *wg*^+^ and *hh*^+^ domains are present, but the Optix^+^ and *dpp*^+^ domains are not yet established. The *Vsx1^+^* domain generates most EONs; the *wg*^+^ tips generate a minority of EONs. Axes as in Fig. 1F. (B) EONs (R9D11-mCD8-GFP^+^, green) are produced from the *Vsx1^+^* domain (red) of the neuroepithelium (outlined). Arrow indicates EON generation. Maximum intensity projection of five 1 µm slices in *z*. (C) Neuroepithelial cells (FasII^+^, white) express L(1)sc (red, arrowhead) in close proximity to EONs (green, arrow). (D,D′) L(1)sc^+^ cells (red) in the neuroepithelium (white) have high EGFR signalling, as assessed using the Pnt-GFP reporter (green) (Boisclair Lachance et al., 2014). (E,E′) L(1)sc^+^ cells (red) in the neuroepithelium (white) have low Notch signalling, as assessed using the HLHmγ-GFP reporter (green) (Almeida and Bray, 2005). (B-E′) Single section confocal images.

We found that almost all EONs are produced by the *Vsx1*^+^ (central) domain of the embryonic neuroepithelium, straddling the presumptive dorsal-ventral boundary (Fig. 2A-B, Fig. S2B,B′). These EONs themselves expressed *Vsx1* (Fig. 2B). We observed that the *wg^+^* tips of the neuroepithelium produce a minority of EONs as assessed using *wg*-LacZ, a reporter inserted at the endogenous *wg* locus (Kassis et al., 1992) (Fig. S2C,C′). Thus, we conclude that the central domain, and to a lesser extent the tips of the embryonic neuroepithelium, produces neuroblasts. Interestingly, we found no evidence for *Optix* or *dpp* expression in the embryonic neuroepithelium (Fig. 2A, Fig. S2D-E′), suggesting that these domains become patterned and start to produce neuroblasts later in development.

### The embryonic neuroepithelium expresses transition zone markers

In the larval brain, neuroepithelial cells are transformed into neuroblasts at a transition zone. The transition zone expresses the proneural gene *lethal of scute* [*l(1)sc*] and the microRNA *miR-7* and is regulated by signalling pathways, including the EGFR and Notch pathways (Fig. S3A) (Caygill and Brand, 2017; Egger et al., 2010; Yasugi et al., 2008, 2010). We found discrete regions of L(1)sc expression in the embryonic neuroepithelium that corresponded spatially with EON production (Fig. 2C). L(1)sc^+^ cells exhibited many features of the larval transition zone: they were positive for EGFR signalling (Fig. 2D-D′), had low Notch signalling (Fig. 2E-E′) and expressed *miR-7* (Fig. S3B). Consistent with a neuroepithelium to neuroblast transition, EONs expressed the neuroepithelial cell markers E-Cadherin (E-Cad) and FasII as they were generated but later downregulated expression of these genes (Fig. S3C-D″).

### EONs generate neurons and glia

**Fig. 3.**
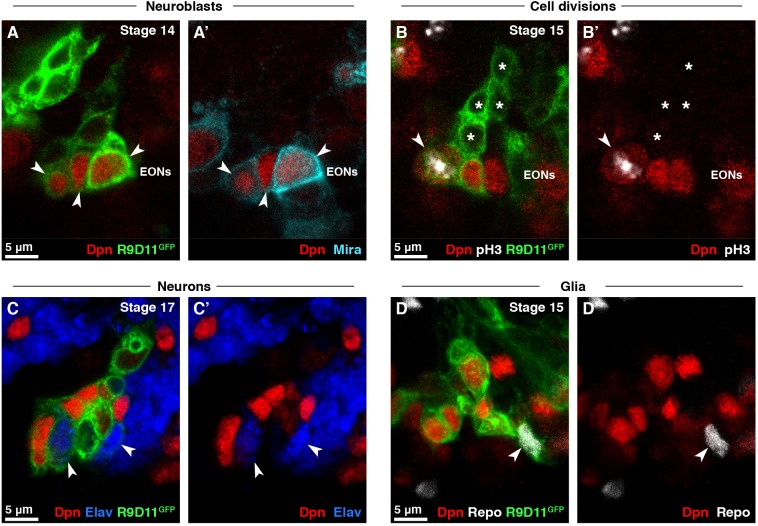
**EONs generate neurons and glia.** (A,A′) EONs (Dpn^+^/R9D11-mCD8-GFP^+^, red and green, arrowheads) express the gene *Mira* (cyan), which is expressed by neuroblasts. (B,B′) EONs (red and green) divide and generate Dpn^−^ progeny (asterisks). Arrowheads indicate a dividing EON, assessed by co-staining for pH3 (white). (C,C′) EONs (red and green) generate Elav^+^ neurons (blue, arrowheads). (D,D′) EONs (red and green) generate Repo^+^ glia (white, arrowheads). Maximum intensity projection of three 1 µm slices in *z*. (A-C′) Single section confocal images.

Neuroblasts in the larval brain divide asymmetrically to generate intermediate progenitor cells (called ganglion mother cells, GMCs) that, in turn, divide once to produce neurons and glia. We found that, like larval neuroblasts, EONs were positive for Wor (Worniu, Fig. S4A,A′) and Mira (Miranda, Fig. 3A,A′), localised Pros (Prospero) and Mira asymmetrically at mitosis (Ikeshima-Kataoka et al., 1997) (Fig. S4B,B′), and divided asymmetrically to generate Dpn^−^ progeny (Fig. 3B,B′). EON lineages were identifiable as R9D11-mCD8-GFP^+^ cells contacting EONs (Fig. 3B,B′). To identify the cell types produced by EONs, we stained for markers specific to GMCs, neurons or glia. We found cells with nuclear Pros (Fig. S4C,C′), Elav (Embryonically lethal abnormal vision, Fig. 3C,C′) or Repo (Reversed polarity, Fig. 3D-D′) next to EONs, corresponding to GMCs, neurons and glia, respectively. By the end of embryogenesis, we found an average of 16.1±1.7 neurons and 3.7±1.4 glia per brain lobe that were in contact with EONs and expressed R9D11-mCD8-GFP (*n*=10 brain lobes).

We confirmed the lineage relationship between EONs and neurons using the FLEXAMP (flip-out LexA amplification) technique, a memory cassette tool (Bertet et al., 2014). We found that neurons were labelled when we expressed FLEXAMP in EONs during embryogenesis (Fig. S4D-E). We conclude that, like canonical neuroblasts, EONs undergo neurogenic divisions and generate differentiated progeny.

### EONs undergo G0 quiescence and persist into the larval brain

At the end of embryogenesis, the majority of neuroblasts in the central brain and ventral nerve cord enter mitotic quiescence or are eliminated by apoptosis (Maurange and Gould, 2005; Truman and Bate, 1988; White et al., 1994). Quiescent neuroblasts persist into the larval brain and later become reactivated in a nutrition-dependent manner to generate neurons and glia in a second round of neurogenesis (Britton and Edgar, 1998; Chell and Brand, 2010; Otsuki and Brand, 2018; Sousa-Nunes et al., 2011; Spéder and Brand, 2014; Truman and Bate, 1988). We assessed whether EONs undergo quiescence or apoptosis at the end of embryogenesis.

**Fig. 4.**
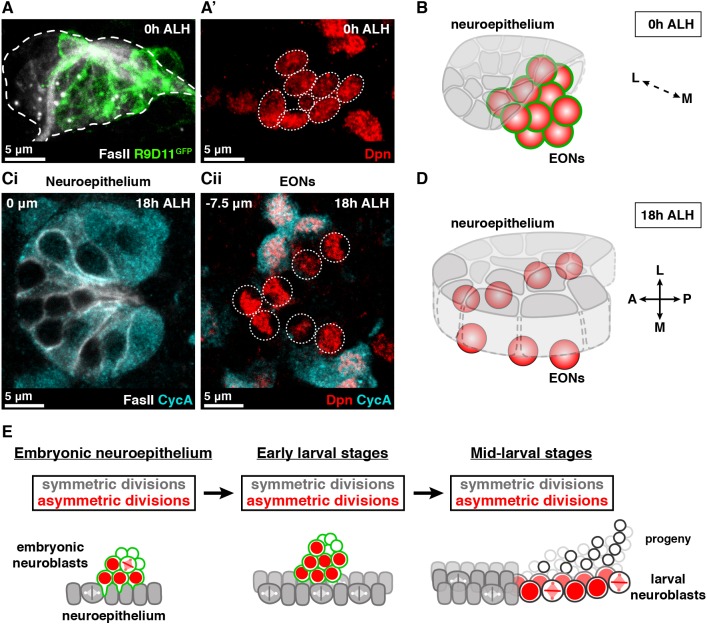
**EONs persist into the post-embryonic brain.** (A,A′) At 0 h ALH, EONs are associated closely with the neuroepithelium (FasII^+^, white and outlined). EONs express R9D11-mCD8-GFP (green) in A and Dpn (red, circled) in A′. Single frame of a 3D reconstruction over a 17 µm confocal stack. The entire 3D reconstruction is available as Movie 1. (B) 3D schematic depicting the spatial relationship between the neuroepithelium and EONs at 0 h ALH. L, lateral; M, medial. (Ci,ii) Single section confocal images taken at indicated depths relative to the neuroepithelium at 18 h ALH. EONs are located medial to the neuroepithelium. (Ci) The neuroepithelium (white) has reactivated and expresses CycA (cyan). (Cii) EONs (red, circled) do not express CycA and are G0 quiescent, in contrast to neighbouring neuroblasts (red and cyan). (D) 3D schematic depicting the spatial relationship between the neuroepithelium and EONs at 18 h ALH. A, anterior; P, posterior; L, lateral; M, medial. (E) Revised model of optic lobe medulla development. Neuroepithelial cells (grey) divide and generate neuroblasts (red and green) in the embryo. After larval hatching, these neuroepithelial cells begin symmetric divisions. From mid-larval stages neuroepithelial cells transform into asymmetrically dividing larval neuroblasts (red and not green). Medial-lateral axis is left-right; brain surface is towards the bottom of the schematic. Compare to Fig. S1A.

We found that EONs persist into the larval brain, identifiable as a cluster of Dpn^+^ R9D11-mCD8-GFP^+^ cells. As in the embryo, EONs are located below the neuroepithelium, medial in the brain (Fig. 4A-B, Movie 1). We observed 10.4±0.6 EONs per brain lobe at 0 h after larval hatching (ALH) (*n*=31 brain lobes), in close agreement with the final number detected in the embryo. The only neuroblasts known to proliferate at larval hatching are the mushroom body and lateral neuroblasts (Ito and Hotta, 1992; Prokop and Technau, 1991; Truman and Bate, 1988), indicating that EONs are quiescent at this stage. It has been shown that quiescent neuroblasts in the brain lobes and ventral nerve cord do not express Wor or Mira (Lai and Doe, 2014; Otsuki and Brand, 2018; Tomancak et al., 2007). In agreement with this, we found that EONs did not express Wor or Mira at 0 h ALH (Fig. S5A-B′), despite expressing these genes previously in the embryo (Fig. 3A,A′, Fig. S4A,A′).

We discovered recently that neuroblasts can undergo two types of quiescence (Otsuki and Brand, 2018). Most quiescent neuroblasts arrest in G2, and only a minority in G0 in the ventral nerve cord. G2 and G0 are two functionally distinct types of stem cell quiescence, as G2 neuroblasts become activated faster than G0 neuroblasts in response to nutritional inputs (Otsuki and Brand, 2018). We found that all EONs undergo G0 quiescence, as they did not express the G2 marker CycA at 0 h ALH (Fig. S5C,C′). We also found that neuroepithelial cells, having divided throughout embryogenesis, eventually become G0 quiescent prior to larval hatching (Fig. S5D). Thus, all neural stem cells in the visual system undergo G0 quiescence, which is otherwise uncommon in the *Drosophila* brain.

### EONs reactivate post-embryonically

The neuroepithelial cells that were generated in the embryo reactivate and begin symmetric divisions during the first larval instar (12-15 h ALH) (Datta, 1995; Nassif et al., 2003). We tested when EONs, which lie below the plane of the neuroepithelium, reactivate. We found that EONs were among the last neuroblasts to reactivate in the brain, consistent with our previous finding that G0 neuroblasts reactivate after G2 neuroblasts (Otsuki and Brand, 2018). EONs were quiescent (small, CycA^−^ and pH3^−^) at 18 h ALH, in contrast to most other neuroblasts in the brain (Fig. 4Ci,ii, D). EONs no longer expressed R9D11-mCD8-GFP at this stage; however, they were readily identifiable based on their position relative to the neuroepithelium. We found that EONs reactivate by 30 h ALH, as all neuroblasts surrounding the neuroepithelium have re-entered the cell cycle (Fig. S5Ei,ii). Thus, we have shown that EONs generate progeny in the embryo, undergo quiescence and become reactivated post-embryonically.

Switches in stem cell division mode are thought to drive the development of both the mammalian cerebral cortex and the *Drosophila* visual system (Fig. S1A). Symmetrically dividing neuroepithelial cells transform into asymmetrically dividing neuroblasts in the *Drosophila* optic lobe during larval development. Here, we have shown that neuroepithelial cells begin to produce neuroblasts in the embryo, demonstrating a much earlier function for both types of neural stem cell in the developing visual system (Fig. 4E). Our discovery that both symmetrically and asymmetrically dividing stem cells are present in the embryo is important given that the mis-regulation of each type of stem cell gives rise to tumours through distinct mechanisms (Bowman et al., 2008; Richter et al., 2011). Our results have implications for understanding the susceptibility of the brain to different types of tumours during embryonic development, with relevance for the progression of childhood tumours (Marshall et al., 2014).

Although embryonic neuroepithelial cells appear to generate neuroblasts in a similar manner to larval neuroepithelial cells, we uncovered several striking differences between the embryonic and larval neuroepithelia. We found that GAL4 drivers commonly used to label the larval neuroepithelium (GAL4*^c855a^* and *ogre*-GAL4) are not expressed in the embryonic neuroepithelium. Larval neuroepithelial cells divide repeatedly and are eventually depleted, in contrast to embryonic neuroepithelial cells that divide once each before becoming quiescent. The larval neuroepithelium produces neuroblasts from all spatial domains, whereas only the *Vsx1^+^* and *wg*^+^ domains produce neuroblasts in the embryo.

Importantly, our results explain recent observations that the larval neuroepithelium expresses L(1)sc, which marks the transition zone, much before the generation of larval neuroblasts (Dillard et al., 2018; Sato et al., 2016). It has been proposed that the transition zone is established at an early stage, ready to induce the neuroepithelium to neuroblast transition later in development (Dillard et al., 2018). Instead, our results demonstrate that L(1)sc expression in the early larval neuroepithelium is a continuation of a neuroepithelium to neuroblast transition that commenced in the embryo.

EONs express R9D11-mCD8-GFP as they are generated by neuroepithelial cells, but later downregulate expression. Intriguingly, we found that R9D11-mCD8-GFP is also expressed at the transition zone in the late larval brain (Fig. S6A). Thus, R9D11-mCD8-GFP expression is common to newly born optic lobe neuroblasts in both the embryo and larva. As R9D11 is a fragment of the *erm* enhancer (Pfeiffer et al., 2008), *erm* might have a function in the transition from neuroepithelial cell to neuroblast.

We have discovered an embryonic phase of neurogenesis originating from the optic lobe neuroepithelium. Although the identities of the neurons born during this embryonic phase are as yet unknown, we find that they lie in close proximity to Bolwig's nerve: part of the larval visual system (Fig. S7A). Tracking the contribution of EONs to the adult brain was not possible in this study because the genetic tools that label EONs, although specific in early development, become widely expressed later in development. The functional contributions of EON lineages to the larval and adult visual systems will be an intriguing topic for future study.

## MATERIALS AND METHODS

### Fly stocks and husbandry

*Drosophila melanogaster* were reared in cages at 25°C, unless indicated otherwise. Embryos were collected onto freshly yeasted apple juice plates overnight and staged according to Campos-Ortega and Hartenstein (1985). For larval experiments, larvae were picked within 1 h of hatching [designated 0 h after larval hatching (ALH)], transferred to a yeasted food plate and reared to the desired stage before dissection.

The following stocks were used: *w^1118^*, GAL4*^c855a^* (Manseau et al., 1997), R9D11-mCD8-GFP (Zhu et al., 2011), R9D11-CD4-tdTomato (Han et al., 2011), (*miR-7*)E>GFP (Li et al., 2009), *wg*-LacZ (1-*en*-11) (Kassis et al., 1992), *hh*^P30^ (Lee et al., 1992) and HLHmγ-GFP (Almeida and Bray, 2005). The following stocks were obtained from the Bloomington *Drosophila* Stock Center: *dpp*-*lacZ^Exel.2^* (#8411), UAS-myr-mRFP (#7119), R31H09-GAL4 (#49694), R29C07-GAL4 (‘*ogre*-GAL4’, #49340) and pnt-GFP.FPTB (#42680). To perform FLEXAMP, we crossed flies carrying *yw*; *tub*-Gal80^ts^, UAS-*flp*; *act*>*y*^+^>LHV2^deltaRFP^-86Fb (LexA) (Yagi et al., 2010) to flies carrying 13XLexAOp2-mCD8-GFP (Bloomington #32205), R31H09-GAL4 and *tub*-GAL80^ts^ (Bloomington #7019).

### Sample fixation

Embryos were washed into a nitex basket with distilled water and dechorionated in 50% bleach/water for 3 min. After rinsing with water, embryos were fixed on a rolling shaker for 20 min in a 6 ml glass bottle containing 3 ml of 4% formaldehyde/PBS and 3 ml heptane. Fixed embryos were washed and stored in methanol at −20°C until ready to immunostain.

Larval brains were dissected in PBS and fixed on a shaker for 20 min in 4% formaldehyde/PBS. Fixed brains were washed well with PBS containing 0.3% Triton-X (PBTx) before immediate immunostaining.

### Immunostaining

Fixed embryos were re-hydrated in 0.3% PBTx and blocked on a shaker for at least 15 min in 10% normal goat serum/PBS. Embryos were incubated overnight at 4°C with primary antibodies diluted in 0.3% PBTx. Embryos were washed well with 0.3% PBTx, then incubated overnight at 4°C with secondary antibodies diluted in 0.3% PBTx. Embryos were washed well with 0.3% PBTx then mounted in 50% glycerol/PBS. Larval brains were processed identically to embryos, with the following alterations: (1) the re-hydration step was omitted and (2) brains were mounted in Vectashield (Vector laboratories).

The following primary antisera were used: mouse 22C10 1:50 (DSHB), chicken anti-βgal 1:1000 (Abcam, ab9361), rabbit anti-CycA 1:100 (Whitfield et al., 1990; rb270), guinea pig anti-Dpn 1:5000 (Caygill and Brand, 2017), rat anti-Dpn 1:100 (Abcam, 11D1BC7, ab195173), rat anti-E-Cad 1:20 (DSHB, DCAD2 conc.), rat anti-Elav 1:100 (DSHB, 7E8A10 conc.), mouse anti-FasII 1:20 (DSHB, 1D4 conc.), chick anti-GFP 1:2000 (Abcam, ab13970), rat anti-Mira 1:500 (a kind gift from C. Q. Doe, University of Oregon, USA), rabbit anti-Optix 1:500 (Kenyon et al., 2005), mouse anti-Pros 1:30 (DSHB, MR1A conc.), rabbit anti-pH3 1:100 (Merck Millipore, 06-570), rat anti-pH3 1:200 (Abcam, ab10543), rabbit anti-Repo 1:10,000 (a kind gift from B. Altenhein, University of Cologne, Germany), guinea pig anti-Vsx1 1:1000 (Erclik et al., 2008) and rat anti-Wor 1:100 (Abcam, 5A3AD2, ab196362). Guinea pig anti-L(1)sc (1:1000) was generated by C. M. Davidson, E. E. Caygill and A.H.B. using constructs that were a kind gift from J. Skeath (Washington University, USA). Primary antibodies were detected using Alexa Fluor-conjugated secondary antibodies (Thermo Fisher Scientific) diluted 1:500 in 0.3% PBTx.

### Lineage tracing with FLEXAMP

To perform FLEXAMP, we crossed flies carrying *yw*; *tub*-Gal80^ts^, UAS-*flp*; *act*>*y*^+^>LHV2^deltaRFP^-86Fb (LexA) to flies carrying 13XLexAOp2-mCD8-GFP, R31H09-GAL4 and *tub*-GAL80^ts^. Embryos were collected for 3 h at room temperature, then raised at 29°C (test) or 18°C (control) until larval hatching. Larval brains were dissected at 0 h ALH and stained for GFP, Dpn, Elav and/or 22C10 as appropriate.

### Image acquisition and processing

Fluorescent images were acquired using a Leica SP8 confocal microscope. Images were analysed using Fiji (Schindelin et al., 2012). Adobe Photoshop was used to adjust brightness and contrast in images. Adobe Illustrator was used to compile figures.

### Quantification and statistical analysis

R was used for statistical analysis. No data were excluded.

## Supplementary Material

Supplementary information
