# Supplementary material for: A newly discovered neural stem cell population is generated by the optic lobe neuroepithelium during embryogenesis in Drosophila melanogaster
Source: Development. 2018 Sep 25;145(18):dev166207. doi: 10.1242/dev.166207 (PMC6176933; doi:10.1242/dev.166207)
Supplement: Supplementary information [file develop-145-166207-s1.pdf]

Supplementary information

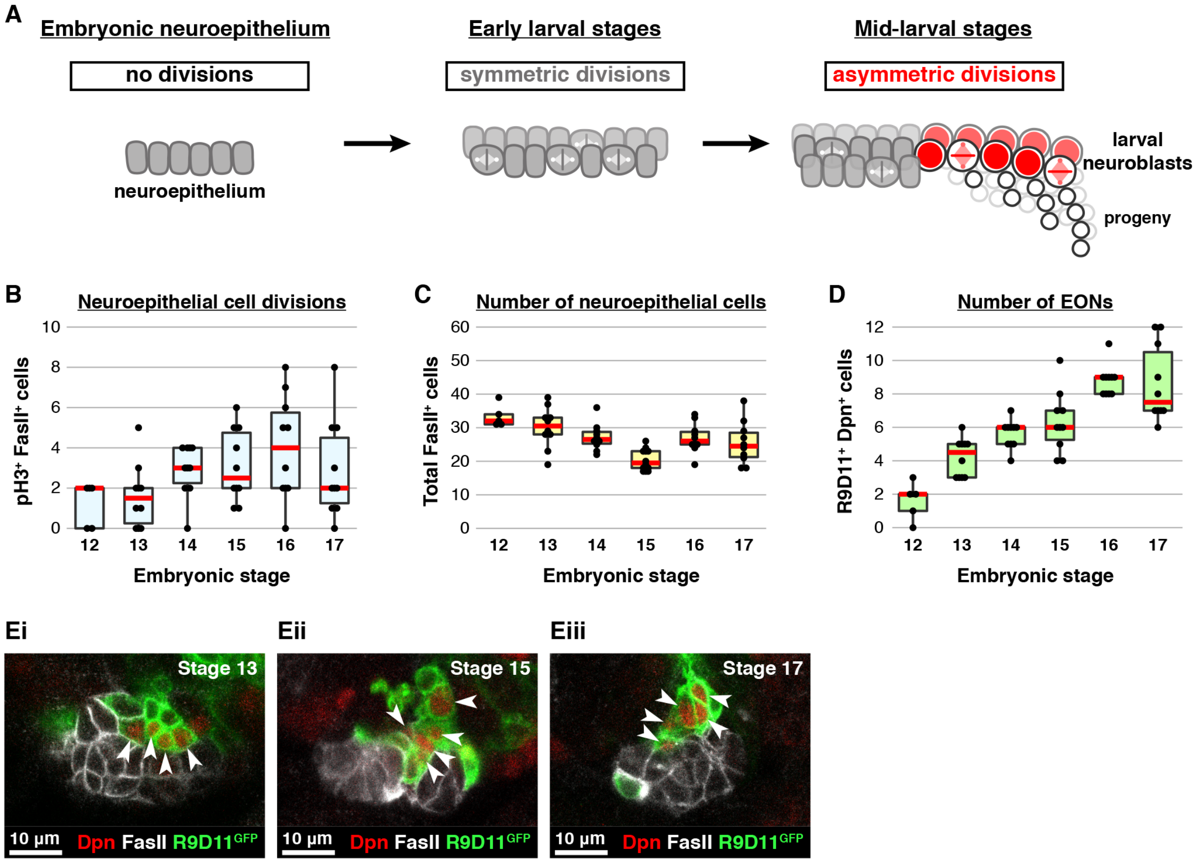

Fig. S1. Neuroepithelial cells divide in the embryo and generate EONs.
(A) During *Drosophila* optic lobe medulla development, neuroepithelial cells (grey) are thought to be quiescent in the embryo. After larval hatching, neuroepithelial cells initiate symmetric, expansive cell divisions. From mid-larval stages, neuroepithelial cells transform into asymmetrically dividing neuroblasts (red), which generate neurons and glia (progeny).
(B) Quantification of dividing neuroepithelial cells per brain lobe between embryonic stages 12 and 17. *n*=10 embryos/stage, except stage 12 for which *n*=5. Red lines indicate medians.
(C) Quantification of neuroepithelial cell number per brain lobe between embryonic stages 12 and 17. *n*=10 embryos/stage, except stage 12 for which *n*=5. Red lines indicate medians.
(D) Quantification of EONs per brain lobe between embryonic stages 12 and 17. *n*=10 embryos/stage, except stage 12 for which *n*=5. Red lines indicate medians.
(Ei-iii) Generation of EONs (arrowheads), labelled with Dpn (red) and R9D11-mCD8-GFP (green), from the embryonic neuroepithelium (FasII$^+$, white). Single section confocal images.

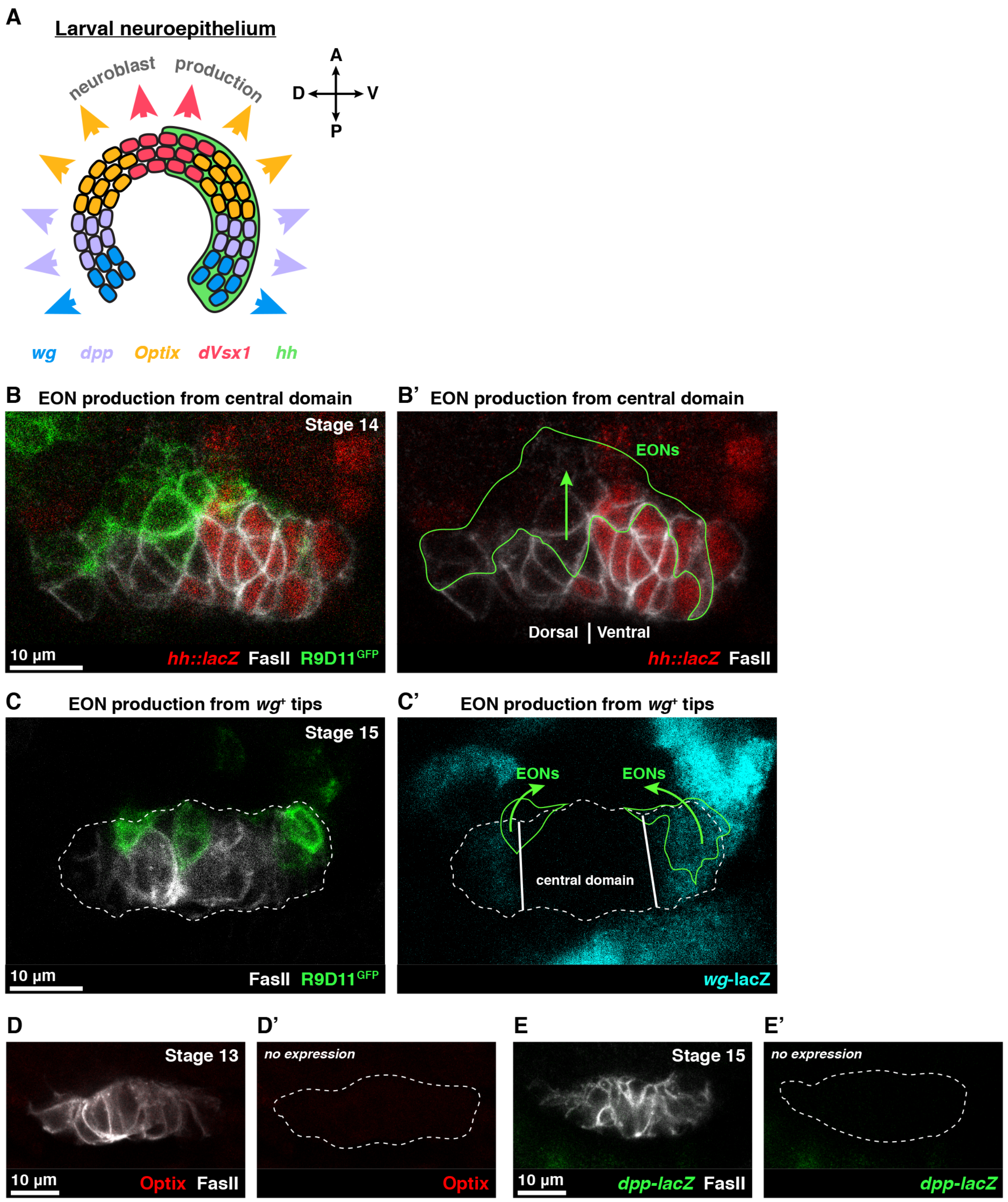

Fig. S2. EONs arise from specific domains of the neuroepithelium.
(A) Spatial patterning domains in the larval neuroepithelium, in lateral view. Anterior is up; posterior is down. Coloured arrows indicate that all domains generate neuroblasts.
(B-B') EONs (R9D11-mCD8-GFP$^+$, green) are generated at the dorsal-ventral boundary of the neuroepithelium (FasII$^+$, white), visualised using *hh::lacZ* (red) (Lee et al., 1992). EONs are outlined and arrow in B' indicates direction of EON production from neuroepithelium.
(C-C') A small number of EONs (green) are generated from the *wg*$^+$ tips of the neuroepithelium (white). *wg*$^+$ domain indicated by expression of *wg*-lacZ (cyan) (Altering the insertional specificity of a Drosophila transposable element., 1992). EONs outlined and arrowed in C'.
(D-D') Neuroepithelial cells (white) do not express Optix (red) in the embryo.
(E-E') Neuroepithelial cells (white) do not express *dpp* in the embryo, assessed using *dpp-lacZ* (green).
Images are single section confocal images, unless indicated otherwise.

Development • Supplementary information

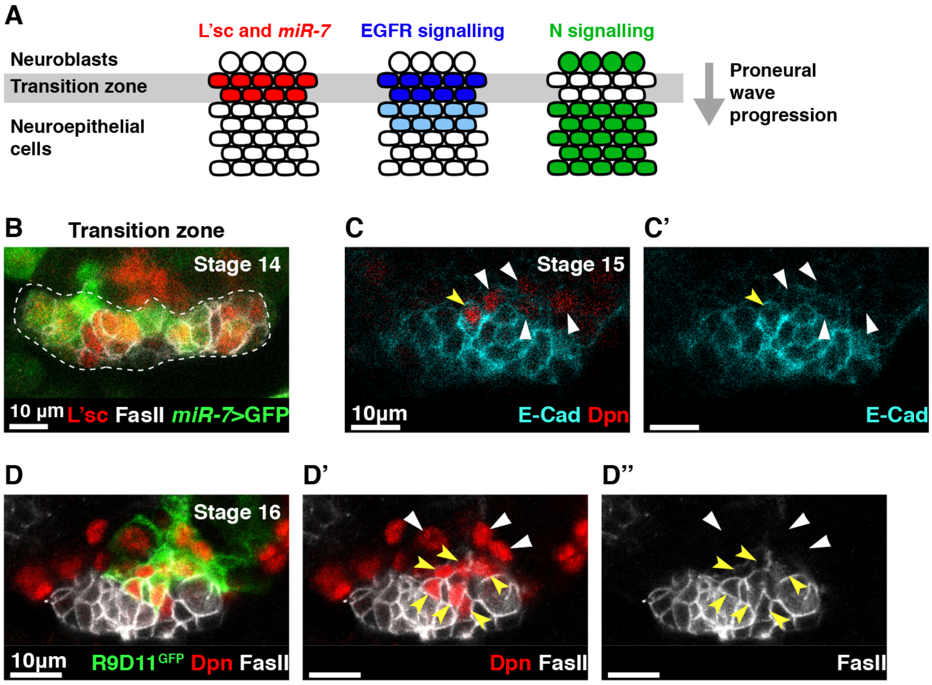

Fig. S3. Embryonic and larval transition zones.
(A) Gene expression and signalling pathways at the larval transition zone. At mid-larval stages transition zone cells express L'sc and *miR-7* (red), have active EGFR signalling (blue) and no Notch signalling (green). Medial is up; lateral is down.
(B) In the embryo, L'sc^{+} cells (red) in the neuroepithelium (FasII^{+}, white and outlined) express *miR-7*, as assessed using the *miR-7*>GFP reporter (green) (Li et al., 2009).
(C-C') E-Cad (cyan) is expressed by neuroepithelial cells and EONs closest to the neuroepithelium (yellow arrowhead). EONs further away from the neuroepithelium downregulate E-Cad expression (white arrowheads).
(D-D'') EONs (red and green in D) closest to the neuroepithelium (yellow arrowheads) express FasII (white). EONs further away from the neuroepithelium (white arrowheads) downregulate FasII.
Single section confocal images.

Development • Supplementary information

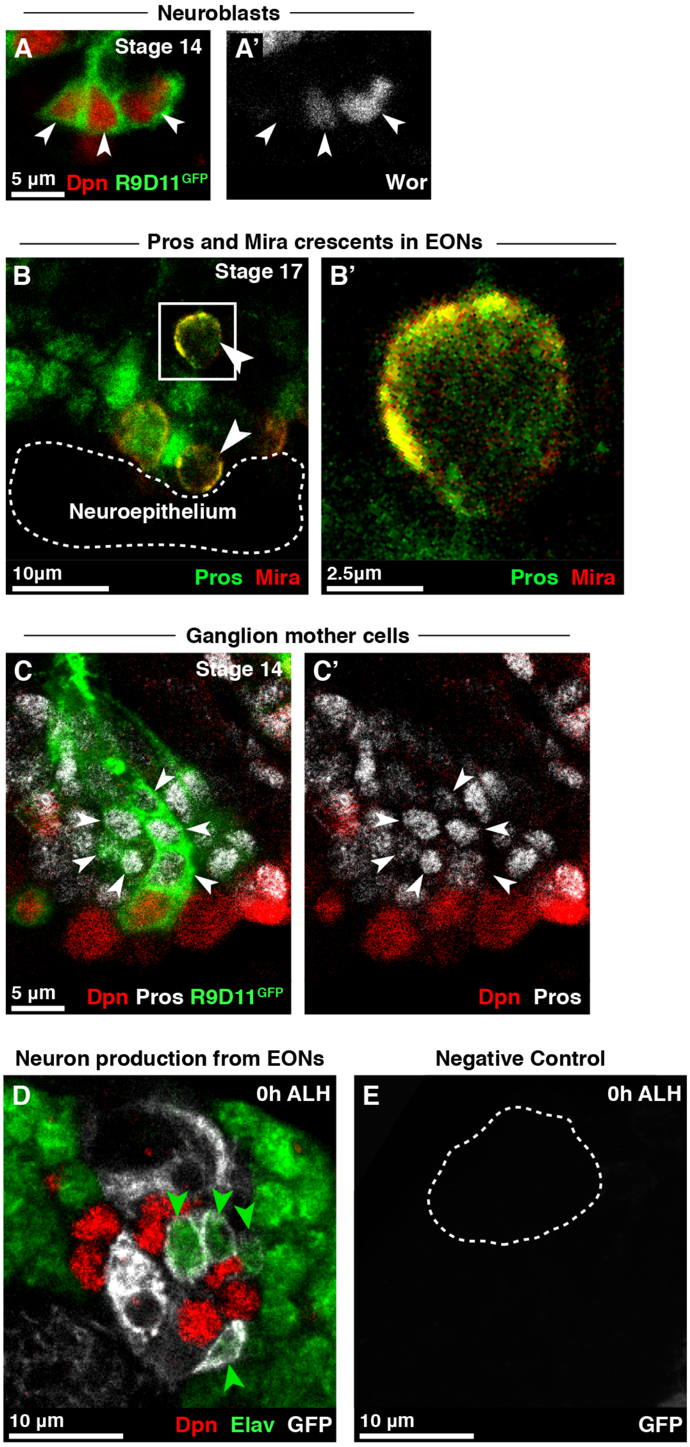

Fig. S4. EONs generate canonical neuroblast lineages.

(A-A') EONs (red and green, arrowheads) express the neuroblast gene Wor (white). Some EONs do not express Wor at this stage and may have already entered quiescence.

(B-B') Arrowheads indicate dividing, which exhibit crescents of Pros (green) and Mira (red). (B') is a magnification of the boxed region in (B).

(C-C') $Pros^{+}$ GMCs (white, arrowheads) are located next to EONs (red and green).

(D) EONs and their progeny were marked by expressing FLEXAMP (white) with R31H09-GAL4. This resulted in labelled neurons (green, arrowed) at 0h ALH.

(E) Negative control for FLEXAMP experiment. Sibling embryos collected from the same cross as in D were raised at 18°C until larval hatching (uninduced FLEXAMP). No signal is observed. Neuroepithelium is outlined.

Single section confocal images.

Development • Supplementary information

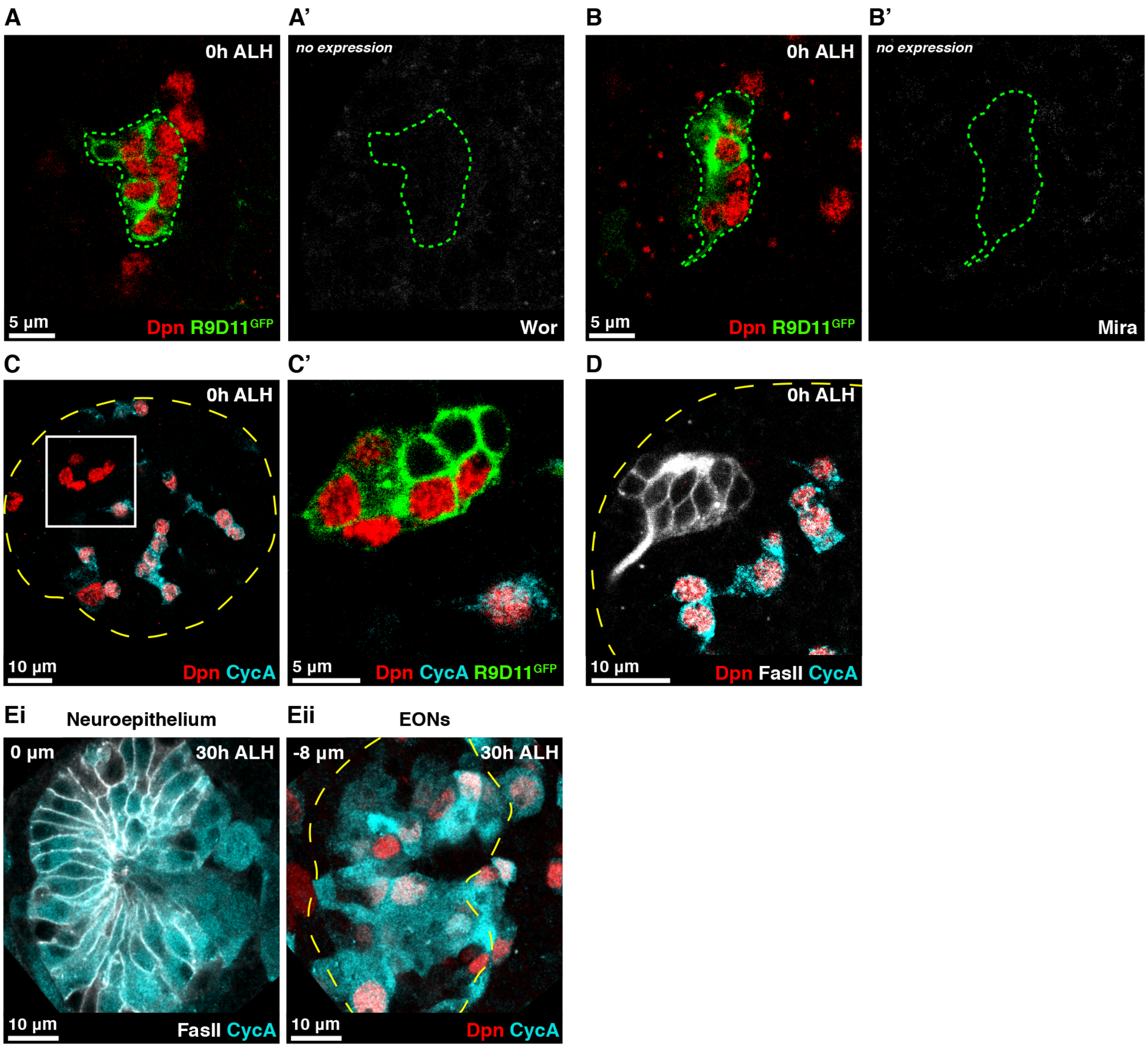

Fig. S5. EONs and neuroepithelial cells undergo quiescence and are reactivated post-embryonically.

(A-A') EONs (Dpn^+/R9D11-mCD8-GFP^+, red and green, outlined) do not express the neuroblast gene Wor (white) at 0h ALH.

(B-B') EONs (red and green and outlined) do not express the neuroblast gene Mira (white) at 0h ALH.

(C-C') EONs (red and green) undergo G_0 quiescence ($CycA^-$). In contrast, the majority of central brain neuroblasts (red) undergo G_2 quiescence ($CycA^+$, cyan). (C') is a magnification of the boxed region in (C). Dashed yellow line outlines the brain lobe.

(D) Neuroepithelial cells ($FasII^+$, white) undergo G_0 quiescence ($CycA^-$) in contrast to neighbouring central brain neuroblasts (red and cyan). Dashed yellow line indicates brain lobe periphery.

(Ei-Eii) EONs (red) have reactivated by 30h ALH, as no quiescent neuroblasts (small, $CycA^-$) are found in the vicinity of the neuroepithelium. Dashed yellow line indicates neuroepithelium periphery.

Single section confocal images.

Development • Supplementary information

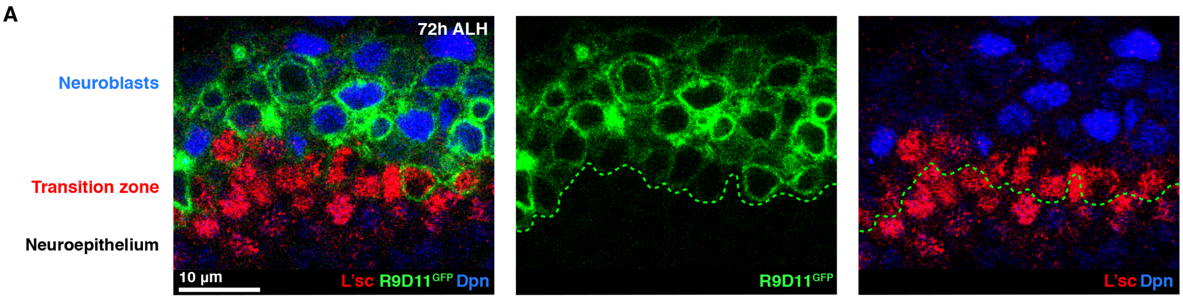

Fig. S6. R9D11-mCD8-GFP is expressed at the larval medulla transition zone.
(A) Larval neuroepithelial cells express R9D11-mCD8-GFP at the transition zone (L'sc^{+}, red) as they become neuroblasts (Dpn^{+}, blue). Green dotted line indicates the front of R9D11-mCD8-GFP expression within the transition zone. Single confocal section.

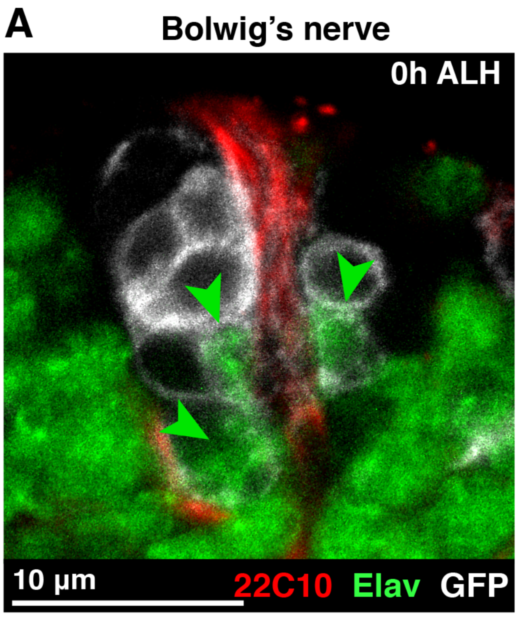

Fig. S7 Neurons produced by EONs directly contact Bolwig's nerve
(A) Green arrowheads indicate neurons (Elav^{+}, green) produced by EONs, which contact Bolwig's nerve (red). EON progeny were labelled using FLEXAMP (white). Elav-negative cells that label with FLEXAMP are neuroepithelial cells and EONs. Maximum intensity projection over 1µm.

Development • Supplementary information

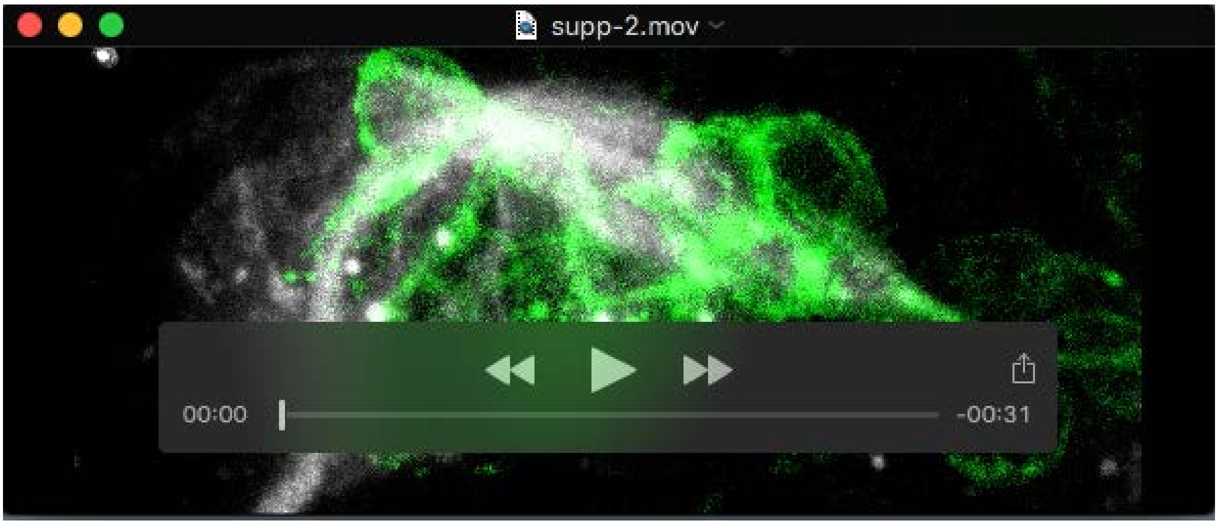

Movie S1. 3D reconstruction depicting the spatial relationship between the neuroepithelium (FasII^{+}, white) and EONs (R9D11-mCD8-GFP^{+}, green) at 0h ALH.
